# Haematological and histopathological monitoring of monosodium glutamate–supplemented feeding in rainbow trout (*Oncorhynchus mykiss*): A possible modelling for a single health approach

**DOI:** 10.1111/jfb.70460

**Published:** 2026-04-28

**Authors:** Gonca Alak, Furkan Bedir, Aslı Çilingir Yeltekin, Serkan Yıldırım, Eylül Ecrin Ekiz, Nisa Gül Yiğit, Esat Mahmut Kocaman, Metin Kiliçlioğlu, Tuba Akbey, Elif Zeynep Özçelik, Koray Koşar, Muhammet Alaeddin Ekiz, Arzu Ucar, Ahmet Atmaca, Melihat Pilgi, Veysel Parlak, Muhammed Atamanalp

**Affiliations:** ^1^ Faculty of Fisheries Atatürk University Erzurum Turkey; ^2^ Faculty of Chemistry Van Yuzuncu Yıl University Van Turkey; ^3^ Veterinary Faculty Ataturk University Erzurum Turkey; ^4^ Veterinary Faculty Kyrgyzstan‐Türkiye Manas University Bishkek Kyrgyzstan; ^5^ Faculty of Erzurum Medicine University of Health Sciences Erzurum Turkey; ^6^ Graduate School of Natural and Applied Sciences Atatürk University Erzurum Turkey; ^7^ Necmeddin Erbakan Science High School Erzurum Turkey

**Keywords:** enzyme activities, feed additive, histomap, oxidative stress

## Abstract

The aim of this study was to investigate the potential effects of monosodium glutamate (MSG), a known flavour enhancer added to many ready‐to‐eat foods, as a feed additive in rainbow trout (*Oncorhynchus mykiss*). Evaluating the effects of MSG on aquatic organisms will fill the knowledge gap in this field by monitoring the haematological indices of O. mykiss with growth parameters and somatic indices, histomap mapping with histopathological observations and determination of antioxidant/cytokine enzyme levels via holistic/multi‐biomarker approaches. Accordingly, rainbow trout feed was supplemented with MSG at different concentrations [(control (0% MSG), MSG‐I (0.25% MSG), MSG‐II (0.5% MSG), MSG‐III (0.75% MSG) and MSG‐IV (1% MSG)], and a 60‐day feeding trial was conducted. Considering the weight gain, MSG‐IV group showed an increase of 24.5% compared to the control and was determined as 61%. Exposure to different concentrations of MSG caused a decrease in erythrocyte, leukocyte, hemoglobin (Hg) and hematocrit (Hct) levels in *O. mykiss*. The effect of the same application on liver tissue, which is the detoxification organ, was determined as inhibition in antioxidant enzyme activities superoxide dismutase (SOD), catalase (CAT), glutathione peroxidase (GPX) and nuclear factor erythroid 2‐related factor 2 (NRF‐2) level, induction in Reactive oxygen species (ROS) ‐malondialdehyde (MDA) level, DNA damage, caspase‐3, tumour necrosis factor α (TNF‐α) and interleukin 6 (IL‐6) activities. Histopathological examination of the liver and intestine revealed hydropic degeneration in a very small number of cells and mild hyperaemia of the blood vessels, with inflammation and DNA damage responses. With the reflection of this situation on histomap results, tissue damage profile and toxicity process differed based on dose and marker and manifested itself with mild‐to‐severe symptoms. These findings revealed that feeding with high concentrations of MSG was effective in NRF‐2/ROS pathways of oxidative stress and showed strong haemato/hepatotoxic effects. Although all MSG concentrations applied were successful in terms of biomass measurements in terms of aquaculture, physiologically, the severity of toxic effects caused by MSG exposure was felt at a low level in liver tissues, and such pollutants should be considered in risk assessment.

## 1 INTRODUCTION

Aquaculture, one of the most dynamic sectors in the global food system, is surprisingly underrepresented in the abundance of literature on food policy. With its high nutritional value, rainbow trout is an important cold‐water fish and is widely harvested worldwide and in Turkey. The increase in aquaculture production has stimulated the demand for fish, and this has also affected the feed sector. Commercial feeds consist of mixtures of different feed ingredients to provide basic nutritional requirements for aquaculture. A wide range of feed additives are used in aquatic feeds to improve the nutritional quality of the diet, increase pellet binding efficiency, prevent the oxidation of sensitive nutrients, increase nutrient availability, eliminate anti‐nutrients, improve product quality and extend shelf life. The development of functional feeds in aquaculture feeds is a new paradigm. Increasing market demand and decreasing supply of fishmeal limit its use as the only source of protein in aquatic feeds (Hossain et al., 2024). Feed additives are substances added in trace amounts to a diet or feed ingredient to improve or protect it. To make the feed more attractive, tasty and digestible products, flavours and digestive aids are added. Often these compounds have little or no nutritional value but are important components of fish feeds, enhancing pellet stability, dietary safety, dietary palatability, fish performance and health status, and affecting the quality of the final product (Yadav et al., 2021).

Formulated feeds and supplements are expected to be economically viable and environmentally friendly, as well as appetizing for fish and not causing any adverse effects on the ultimate consumer – humans. Monosodium glutamate (MSG), which has these properties, is used in the food industry as a flavour enhancer that intensifies the meaty, salty flavour of foods and has a different taste called umami (also known as the fifth basic taste), just as naturally occurring glutamate does in foods, which makes this product suitable for the aquaculture sector.

In the literature, the first MSG‐related studies on fish have been reported since 2018 and were conducted on carps (Zhelyazkov, 2018). In another study, blood biochemistry, feed evaluation and weight gain were determined by adding different amounts of MSG to rainbow trout diets (Zhelyazkov & Stratev, 2019). These studies were followed by catfish and zebrafish (Ladeira et al., 2021; Ngaddi et al., 2019; Sajjaviriya et al., 2019; Suharman et al., 2023).

Considering the above study findings, high doses (2000–4000 mg/kg body weight) of daily MSG intake have been reported to be toxic to humans and experimental animals. The fact that there is only one study on this phenomenon in *Oncorhynchus mykiss*, and that the findings of this research are very limited, as well as the high concentrations used and the short feeding period, revealed that there is an urgent need to investigate additional toxicity pathways. Research using an indicator organism/target tissue/biomarker approach is key to determining the risks of MSG as a potential feed additive in aquatic ecosystems. The most important aim of this study is to determine how MSG will affect the physiology and health of fish, and how it will affect each organ‐tissue where it will show the potential for ultimate target accumulation like a Trojan horse. In this sense, can MSG be used as a cheap and useful feed additive in trout? Considering the multi‐biomarker results, what should be the most appropriate concentration that will not cause tissue‐based damage in the holistic mechanism? is the question of the study design.

## 2 METHODS

### 2.1 Trial components and design

#### 2.1.1 Fish material

Two hundred and twenty‐five rainbow trout (*O. mykiss*), with an average weight of 129 ± 2.5 g obtained from Atatürk University (Erzurum, Turkey) Faculty of Fisheries, Inland Fisheries Research and Application Center, were used as fish material.

#### 2.1.2 Monosodium glutamate

This compound is highly soluble in water and is licensed as E 261 in the E list of food additives (European Union); the use of glutamic acid and its salts in all kinds of food products are specified as 10 mg/kg foodstuff (Dal et al., 2017). Concentrations of MSG to be added to the feeds were determined for 100 g fish weight, considering human consumption levels, and this additive was obtained from a commercial company.

#### 2.1.3 Feed material and feed production

Feed material (fish meal, fish oil, soybean by‐products, wheat by‐products, wheat by‐products, vitamins and minerals with a chemical composition of 45% crude protein, 20% crude fat, 3% crude cellulose, 12% moisture, 12% ash, 5090 kcal/kg gross energy, 4430 kcal/kg digestible energy) was obtained from a commercial company. After the commercial feeds were ground into flour (for MSG addition) in grinders, MSG was added as feed additive at the determined levels (0%, 0.25%, 0.5%, 0.75% and 1%). Feed raw materials were mixed using a laboratory‐type mixer, to ensure homogeneity. The pelleted feeds [control (0% MSG), MSG‐I (0.25% MSG), MSG‐II (0.5% MSG), MSG‐III (0.75% MSG) and MSG‐IV (1% MSG)] were dried in an oven at 135°C for 2 h and fractionated based on the mouth openings of the fish (Lunger et al., 2007). The fractionated feeds were placed in airtight glass boxes and stored at −20°C away from light. The feed was characterized using Fourier‐transform infrared spectroscopy (FT‐IR) with a PerkinElmer Spectrum Two spectrometer (Perkin Elmer, MA, USA) (Yıldırım et al., 2025). Fish were fed with this feed for 2 months. During the experiment, pH, temperature and dissolved oxygen content, which are important water quality parameters in terms of feeding, were monitored daily.

#### 2.1.4 Trial design

In the study, five groups (one control and four treatments) were formed, and fish (according to OECD 210 report) were randomly distributed into the aquariums in three replicates, with 15 fish in each group (Turkez et al., 2025). The fish were acclimated in the same conditions before the trial for 14 days. The daily amounts of the dried feeds were designed by considering the water temperature and fish weight, and the feeding programme was continued by recalculating the daily feed amounts for 15‐day periods by calculating the 1/1 weight gain after 3 days of feeding. Biomass measurements were made at certain intervals (0, 15, 30, 45 and 60 days) during the feeding trial (2 months), and feeding tables were reconstructed. The feeding programme was calculated for each diet based on water temperature (10 ± 1°C) and fish weight and was administered twice daily, in the morning and afternoon (Table S1) (Atamanalp et al., 2023).

#### 2.1.5 Ethics statement

The care and use of experimental animals complied with Atatürk University Animal Experimental and Ethical Committee animal welfare laws, guidelines and policies under approval number E‐75296309‐050.01.04‐2500167039/2025/06 and 2025/06. The study was approved by the committee in Erzurum, Turkey.

### 2.2 Biometric analysis

In the study, total body weight, net growth, daily specific growth, feed conversion ratio (FCR), viscerosomatic index (VSI) and hepatosomatic index (HIS) values of the fish were determined at 15‐day periods from the beginning of the experiment. Parameters were calculated according to Kocaman et al. (2024) (see Supplementary file).

### 2.3 Determination of hematologic indices

In blood samples (seven fish/group) collected at monthly intervals, haematological parameters such as erythrocyte count (RBC), leukocyte count (WBC), haemoglobin value (Hb), haematocrit ratio (Hct), platelet count (PLT), mean corpuscular haemoglobin concentration (MCHC), mean corpuscular haemoglobin (MCH) and mean corpuscular volume (MCV) were determined. To obtain fast and reliable results, readings were taken using a Prokan brand haematology analyser PE‐6800VET model fully automatic blood counting device calibrated based on fish species (Alak et al., 2023). Due to waiting time, only the erythrocyte sedimentation rate (ESR) parameter was determined manually. For the determination of ESR, anticoagulated blood samples were taken into haematocrit tubes with a diameter of 1.1–1.2 mm and a length of 7 cm and kept in an upright position (90°) for 1 h, and the serum separated at the end of the time was measured with a ruler and recorded in mm/h (Alak et al., 2018).

### 2.4 Determination of oxidative stress response and cytokine enzyme levels

This analysis was scanned and interpreted at monthly intervals during the feeding programme (60 days). The amount of KH_2_PO_4_ buffer solution was added thrice to the liver sample taken from the fish, and the homogenized homogenates were centrifuged (13,000 rpm, 30 min 4°C), and the supernatant was taken, and direct enzyme activity measurements were performed (Alak et al., 2023). An ELISA kit was used to determine enzyme activities, lipid peroxidation, apoptosis, DNA damage and cytokine enzyme levels, and the procedure specified in the kit was applied for each parameter (Alak et al., 2023; Atamanalp et al., 2023) (Table S2).

### 2.5 Histopathological examinations

This analysis was scanned and interpreted at monthly intervals during the feeding programme (60 days). For this purpose, tissue samples taken from the liver and gastrointestinal tract of five randomly sampled fish were fixed in 10% formalin solution for 48 h and embedded in paraffin blocks for histopathological examinations after routine tissue follow‐up procedures, and sections (4 μm thick) were taken from these blocks. The preparations for histopathological examination were stained using haematoxylin–eosin (HE) and examined by light microscopy (Olympus, BX 51, JAPAN) for scoring. Sections were evaluated as absent (−), mild (+), moderate (+++) and severe (++++) according to histopathological findings) (Kocaman et al., 2024).

### 2.6 Statistical analysis

Growth performance parameters, haematological index, antioxidant and cytokine enzymes, DNA damage and apoptosis data, were analysed separately for each feeding period or sampling time. Within each time interval, differences among dietary treatment groups were tested using one‐way analysis of variance (ANOVA) under the general linear model (GLM) procedure. Before analysis, data were checked for normality and homogeneity of variances. When significant differences were detected (*p* < 0.05), post hoc multiple‐comparison tests were applied to identify differences among groups. GraphPad Prism 8.0.2 programme was used for statistical analysis in histopathological examinations, and data were evaluated by accepting *p* < 0.05 as significant. Non‐parametric Kruskal–Wallis test was used to determine group interaction, and Mann–Whitney *U* test was used to determine the differences between groups.

## 3 RESULTS

**FIGURE 1:**
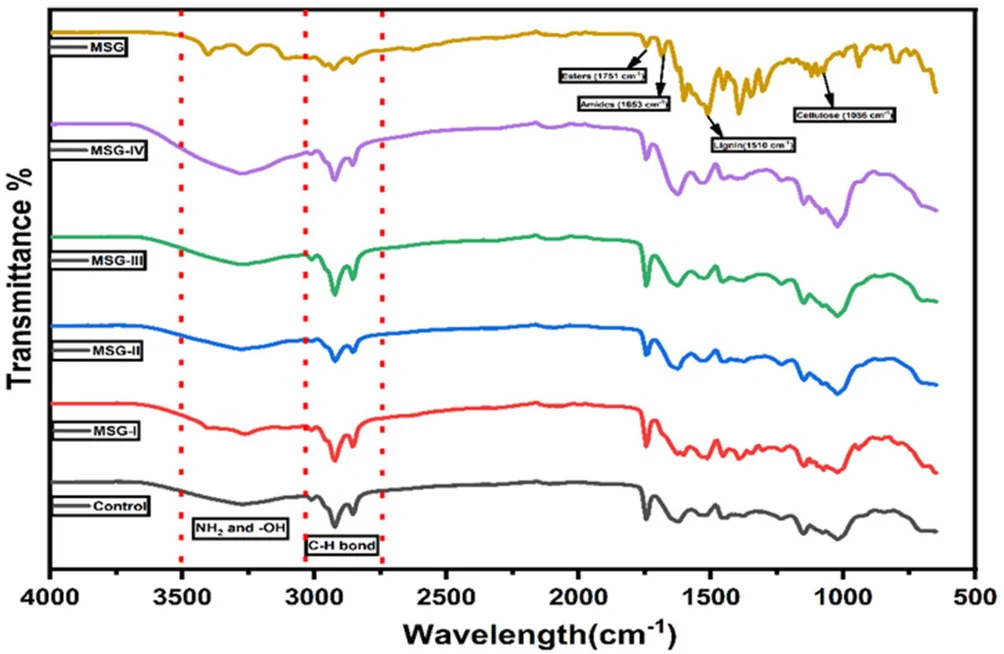
Fourier‐transform infrared spectroscopic (FT‐IR) image of synthesized substances.

In prepared feeds, according to the FT‐IR spectra, the control, MSG‐I, MSG‐II, MSG‐III, MSG‐IV and PURE MSG samples are identified as shown in Figure 1.

**FIGURE 2:**
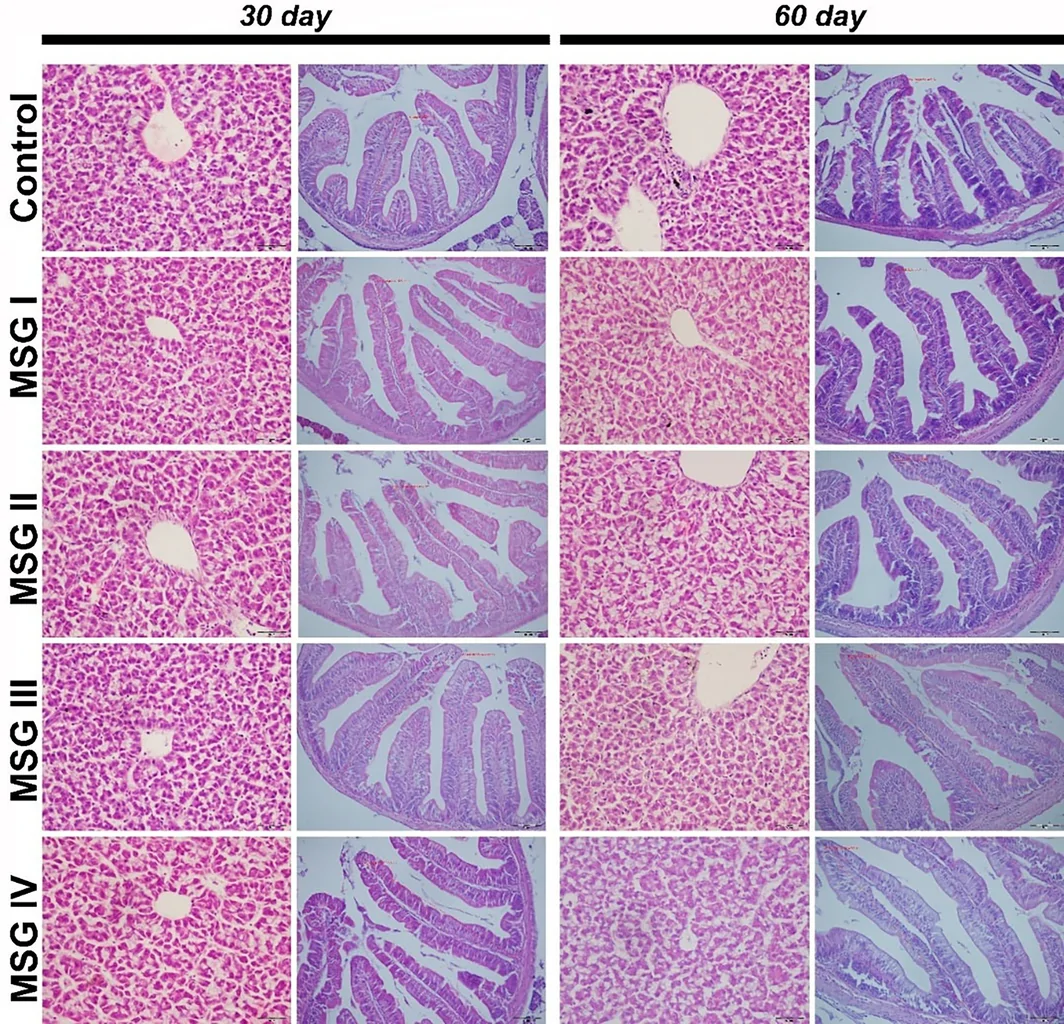
Liver and intestinal tissue, haematoxylin–eosin (H&E). Bar: 100 μm.

**FIGURE 3:**
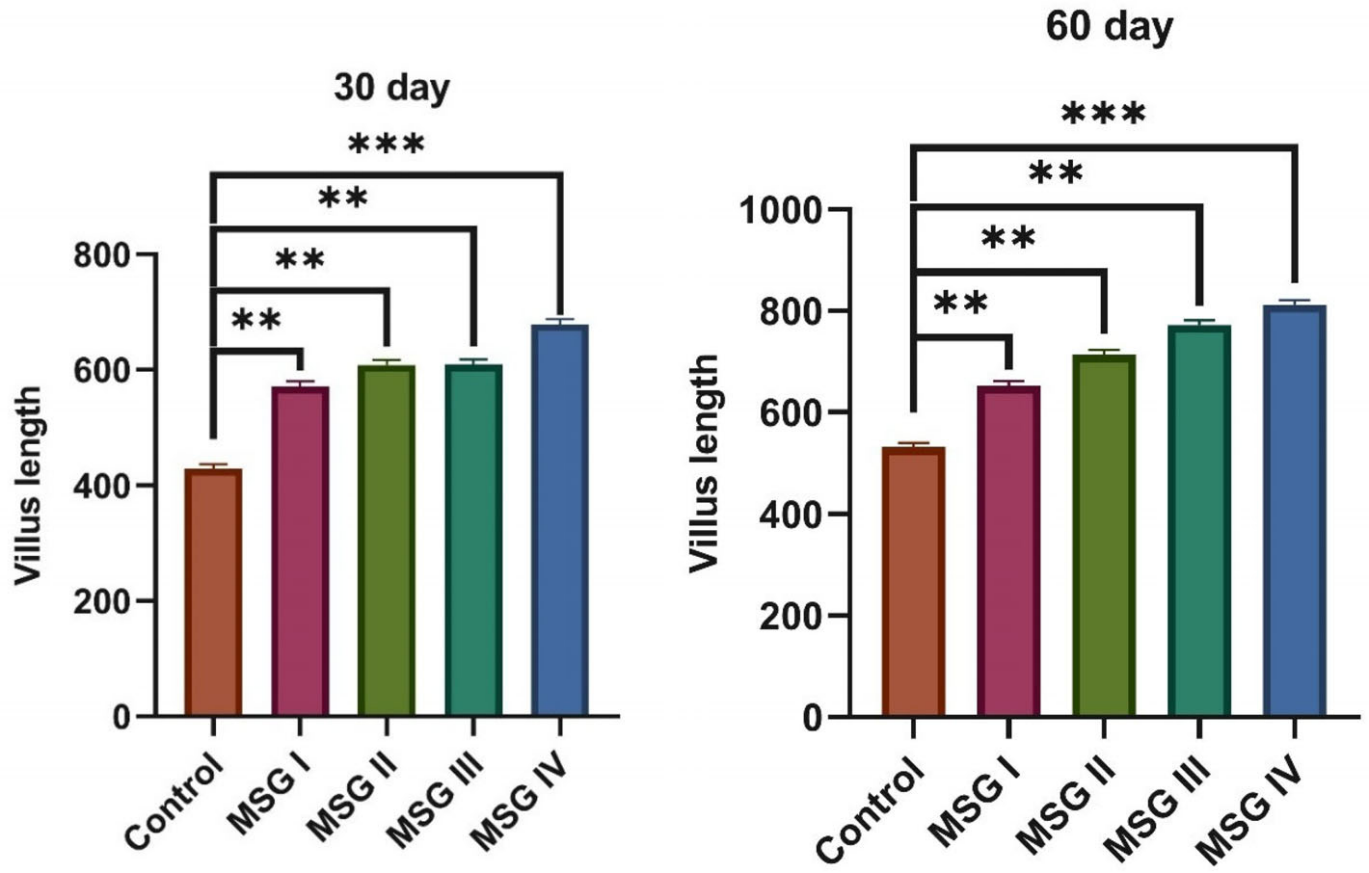
Statistical analysis data of intestinal villus lengths (**p = 0.0022, ***p < 0.0001).

Our findings showed that the addition of MSG decreased the FCR and increased the weight of the fish, but no mortality occurred (Table 1). Considering the weight gain, MSG‐IV group showed an increase in 24.5% compared to the control and was determined as 61%. In the first period of the feeding trial, HSI values were 1.29 ± 0.23, 1.83 ± 0.02, 1.81 ± 0.17, 1.94 ± 0.45 and 2.09 ± 0.70 for control, MSG‐I, MSG‐II, MSG‐III and MSG‐IV, respectively; however, these increases were not statistically significant. In contrast, VSI values were 9.68 ± 2.44, 10.27 ± 3.38, 11. 59 ± 1.01, 12.41 ± 4.23 and 14.07 ± 1.48, respectively. At the end of the experiment period, increases in the same index values were recorded as MSG‐IV>MSG‐III>MSG‐II>MSG‐I>control (HSI values 2.02 ± 0.041 > 1.99 ± 0.47 > 1.95 ± 0.59 > 1.91 ± 0.73 > 1, 1.81 ± 0.24), and VSI was determined as MSG‐IV > MSG‐III > MSG‐II > MSG‐I > control (13.22 ± 3.64 > 13.11 ± 0.82 > 12.82 ± 1.76 > 11.54 ± 0.50 > 10.47 ± 1.95), but these relative changes were not statistically significant (*p* > 0.05). Considering the histopathological findings, it was determined that liver tissues in the control, MSG‐I, MSG‐II and MSG‐III groups showed normal histological appearance during the first part of the feeding period (day 30) (Figure 2). However, in the MSG‐IV group, hydropic degeneration was observed in very few liver cells and mild hyperaemia in the vessels; however, these differences were not statistically significant (*p* > 0.05) (Figure 2). In intestinal tissues, normal histologic structure was found in control, MSG‐I and MSG‐II groups. However, in MSG‐III and MSG‐IV groups, intestinal tissues were found to have a normal histologic structure (Figure 2), and intestinal villus lengths showed a statistically significant (*p* < 0.05) difference compared to the control group (Figures 2 and 3). The necroscopy results at the end of the feeding trial showed that the liver tissue in the control and treatment groups (MSG‐I, MSG‐II and MSG‐III) had a normal histologic appearance (Figure 2). However, degeneration was observed in very few hepatocytes in MSG‐IV group liver tissue, but not at a statistically significant level (Figure 3). In the intestinal histomap images, it was found that the intestinal tissues in the control and treatment groups had a normal histologic structure, and the changes in the intestinal villus lengths in MSG‐II, MSG‐III and MSG‐IV groups were statistically significant (*p* < 0.05) compared to the control group (Figure 3).

**TABLE 1:** Biomass data for the feeding trial.

Feeding time (days)	groups	Growth parameters					
		Daily growth (DG)	Feed conversion ratio (FCR)	Specific growth rate (SGR)	Total weight gain (TWG)	Mean weight gain (MWG)	Survival rate (SR)
0–15	Control	18.00 ± 0.66a	0.75 ± 0.03a	1.08 ± 0.03a	270.00 ± 10.0a	22.49 ± 0.83a	No mortality
	MSG‐I	19.33 ± 0.67a	0.71 ± 0.01a	1.14 ± 0.02a	290.00 ± 10.0a	24.16 ± 0.83a	No mortality
	MSG‐II	20.67 ± 0.66a	0.66 ± 0.02a	1.21 ± 0.04a	310.00 ± 10.0a	25.83 ± 0.83a	No mortality
	MSG‐III	20.66 ± 2.00a	0.67 ± 0.06a	1.19 ± 0.10a	310.00 ± 30.0a	25.83 ± 2.50a	No mortality
	MSG‐IV	21.94 ± 1.03a	0.99 ± 0.48a	1.06 ± 0.48a	270.00 ± 13.0a	22.49 ± 10.8a	No mortality
15–30	Control	15.44 ± 3.33b	1.05 ± 0.23 b	0.78 ± 0.15 b	230.00 ± 50 b	19.16 ± 4.16 b	No mortality
	MSG‐I	18.66 ± 2.66ab	0.82 ± 0.12 ab	0.96 ± 0.13 ab	280.00 ± 40 a	23.44 ± 3.33 ab	No mortality
	MSG‐II	18.27 ± 0.66ab	0.82 ± 0.01 ab	0.94 ± 0.01 ab	280.33 ± 0.57 ab	23.32 ± 0.02 ab	No mortality
	MSG‐III	18.71 ± 5.33ab	0.87 ± 0.19 ab	0.94 ± 0.19 ab	280.00 ± 80 ab	23.44 ± 6.67 ab	No mortality
	MSG‐IV	23.44 ± 0.66a	0.67 ± 0.03 a	1.13 ± 0.04 a	350.00 ± 10 a	29.16 ± 0.83 a	No mortality
30–45	Control	9.33 ± 4ab	1.45 ± 0.32ab	0.46 ± 0.10b	156.67 ± 66.58a	12.77 ± 3.89a	No mortality
	MSG‐I	8.67 ± 3.33b	1.52 ± 0.35a	0.44 ± 0.09b	130.00 ± 50a	11.94 ± 3.06a	No mortality
	MSG‐II	11.99 ± 1.33ab	1.07 ± 0.04bc	0.59 ± 0.02ab	180.00 ± 20a	17.14 ± 0.56a	No mortality
	MSG‐III	11.55 ± 2.03ab	1.06 ± 0.05bc	0.56 ± 0.02ab	171.67 ± 30.13a	16.17 ± 0.56a	No mortality
	MSG‐IV	15.44 ± 4.67a	0.83 ± 0.14c	0.71 ± 0.12a	230.00 ± 70a	18.25 ± 1.15a	No mortality
45–60	Control	7.42 ± 4.67a	1,58 ± 1.12a	0.33 ± 0.14c	110.00 ± 70b	9.81 ± 5.28c	No mortality
	MSG‐I	8.78 ± 3.34a	1.40 ± 0.34b	0.41 ± 0.08bc	133.33 ± 50.33b	11.96 ± 3.06bc	No mortality
	MSG‐II	12.77 ± 2.00a	1.14 ± 0.05ab	0.58 ± 0.02ab	191.67 ± 30.13ab	18.10 ± 0.29ab	No mortality
	MSG‐III	18.20 ± 6.55a	0.99 ± 0.08b	0.68 ± 0.13a	243.33 ± 80.20a	22.47 ± 4.46a	No mortality
	MSG‐IV	44.22 ± 5.70a	0.74 ± 0.26b	0.54 ± 0.03ab	173.33 ± 30.55ab	16.07 ± 0.55abc	No mortality
0–60	Control	12.33 ± 3.67ab	1.23 ± 0.29a	0.62 ± 0.14b	740.00 ± 22ab	61.66 ± 18.3ab	No mortality
	MSG‐I	12.16 ± 0.83b	1.16 ± 0.06ab	0.64 ± 0.03ab	730.00 ± 50b	60.83 ± 4.17b	No mortality
	MSG‐II	14.33 ± 1.33ab	1.01 ± 0.11ab	0.70 ± 0.04ab	860.00 ± 80ab	71.66 ± 6.66ab	No mortality
	MSG‐III	15.83 ± 1.17ab	0.91 ± 0.07b	0.76 ± 0.06ab	950.00 ± 70ab	79.16 ± 5.83ab	No mortality
	MSG‐IV	16.16 ± 1.50a	0.92 ± 0.09b	0.78 ± 0.05a	970.00 ± 90a	80.83 ± 7.50a	No mortality

*Note*: Different lowercase letters within the same column and within the same sampling time indicate significant differences among treatment groups (*p* < 0.05) (mean ± standard error).

In the monthly haematological index data obtained at the end of the 60‐day feeding trial with MSG, decreases were observed in RBC, WBC, Hg, Hct and MCHC levels with increasing dose, whereas increases were observed in MCV and MCH levels (Table 2). However, these changes were not statistically significant.

**TABLE 2:** Haematological indexes.

Feeding time (days)	Groups	WBC	RBC	HGB	HCT	MCV	MCH	MCHC	PLT	ESR
30	Control	19.23 ± 2.20a	2.29 ± 0.40a	14.92 ± 2.59a	38.43 ± 6.16a	168.05 ± 5.82a	64.95 ± 2.13ab	38.70 ± 1.19a	780.33 ± 22.63a	0.10 ± 0.00a
	MSG‐I	17.35 ± 1.79a	2.25 ± 0.22a	14.58 ± 1.92a	38.70 ± 4.10a	171.63 ± 2.15a	64.45 ± 2.68ab	37.55 ± 1.34a	733.17 ± 16.48a	0.13 ± 0.05a
	MSG‐II	16.82 ± 1.53a	2.04 ± 0.24a	12.98 ± 3.01a	35.38 ± 6.08a	173.33 ± 13.11a	63.05 ± 7.67b	36.40 ± 3.42a	731.67 ± 19.49a	0.23 ± 0.01a
	MSG‐III	16.83 ± 3.91a	2.00 ± 0.47a	14.16 ± 1.95a	34.90 ± 9.74a	172.88 ± 13.08a	73.56 ± 14.76a	43.55 ± 13.24a	529.67 ± 22.20a	0.23 ± 0.02a
	MSG‐IV	15.72 ± 3.90a	1.97 ± 0.77a	12.75 ± 5.18a	33.28 ± 13.37a	167.93 ± 4.16a	64.68 ± 2.99ab	38.62 ± 2.24a	527.33 ± 23.91a	0.27 ± 0.02a
60	Control	22.26 ± 2.90a	2.28 ± 0.41a	16.87 ± 7.74a	38.40 ± 3.10a	168.73 ± 8.86a	76.40 ± 11.80a	45.16 ± 6.68a	677.00 ± 69.84a	0.13 ± 0.05a
	MSG‐I	20.76 ± 3.45a	2.23 ± 0.82a	15.03 ± 3.10a	34.76 ± 6.63a	157.40 ± 9.97a	71.16 ± 8.49a	45.03 ± 4.29a	505.33 ± 28.78a	0.17 ± 0.11a
	MSG‐II	19.96 ± 2.31a	2.16 ± 0.52a	15.03 ± 3.47a	36.00 ± 7.45a	167.00 ± 4.21a	70.00 ± 4.78a	41.93 ± 1.91a	453.33 ± 50.81a	0.17 ± 0.05a
	MSG‐III	18.10 ± 1.53a	2.13 ± 0.78a	15.73 ± 2.85a	36.00 ± 4.40a	168.00 ± 6.10a	79.30 ± 11.44a	47.66 ± 7.28a	473.67 ± 37.09a	0.17 ± 0.05a
	MSG‐IV	17.83 ± 2.24a	1.83 ± 0.76a	13.86 ± 1.64a	31.16 ± 6.18a	172.23 ± 7.12a	85.50 ± 15.42a	49.16 ± 9.16a	398.67 ± 38.47a	0.20 ± 0.10a

*Note*: Different lowercase letters within the same column and within the same sampling time indicate significant differences among treatment groups (*p* < 0.05) (*n* = 7*3, mean ± standard deviation). *WBC (104/mm3), *RBC (106/mm3), *Hb (g/dL), Htc (%), *PLT (104/mm3), *MCV (μm^3^), *MCH (pg), *MCHC (g/100 mL), *ESR (mm/h). Abbreviations: ESR, erythrocyte sedimentation rate; Hb, haemoglobin value; Hct, haematocrit ratio; MCH, mean corpuscular haemoglobin; MCHC, mean corpuscular haemoglobin concentration; MCV, mean corpuscular volume; PLT, platelet count; RBC, red blood cells; WBC, white blood cells.

In our study, antioxidant enzyme activities (superoxide dismutase [SOD], catalase [CAT] and glutathione peroxidase [GPx]), as well as cytokine enzyme NRF‐2 level, decreased, whereas reactive oxygen species (ROS), MDA, IL‐6, tumour necrosis factor α (TNF‐α), DNA damage and apoptosis markers were highly expressed in the liver tissue of rainbow trout fed with MSG compared to the control during the feeding trial. At the end of the feeding period (day 60), SOD, CAT, GPx, NRF‐2, MDA, ROS, caspase‐3 and 8‐OHdG levels were found to be statistically significant (*p* < 0.05), whereas IL‐6 and TNF‐α were found to be statistically insignificant at *p* < 0.05 level, although increasing trends were recorded (Table 3).

**TABLE 3:** Effect of monosodium glutamate (MSG) on liver's antioxidant and cytokine enzymes, DNA damage and apoptosis of rainbow trout.

Feeding time (days)	Groups										
		SOD	CAT	GSH‐px	ROS	MDA	NRF‐2	TNF‐α	IL‐6	8‐OHdG	Caspase‐3
30	Control	0.34 ± 0.05a	8.54 ± 0.26a	0.35 ± 0.06a	0.13 ± 0.00a	22.70 ± 4.01a	27.21 ± 2.72a	12.89 ± 1.12^a^	2.75 ± 0.03 ^b^	1.03 ± 0.11b	0.85 ± 0.01d
	MSG‐I	0.26 ± 0.05a	8.39 ± 0.26a	0.27 ± 0.06a	0.15 ± 0.00a	26.16 ± 4.01a	25.96 ± 2.72a	13.46 ± 1.12^a^	2.73 ± 0.03 ^b^	1.15 ± 0.11ab	0.86 ± 0.01 cd
	MSG‐II	0.23 ± 0.05a	8.28 ± 0.26a	0.26 ± 0.06a	0.18 ± 0.00a	26.94 ± 4.01a	22.76 ± 2.72a	14.02 ± 1.12 ^a^	2.74 ± 0.03 ^b^	1.17 ± 0.11ab	0.89 ± 0.01c
	MSG‐III	0.21 ± 0.05a	8.24 ± 0.26a	0.25 ± 0.06a	0.19 ± 0.00a	28.63 ± 4.01a	22.22 ± 2.72a	14.13 ± 1.12^a^	2.74 ± 0.03 ^b^	1.29 ± 0.11ab	0.92 ± 0.01b
	MSG‐IV	0.20 ± 0.05a	8.12 ± 0.26a	0.21 ± 0.06a	0.30 ± 0.00a	29.40 ± 4.01a	22.15 ± 2.72a	14.40 ± 1.12^a^	2.92 ± 0.03 ^a^	1.47 ± 0.11a	0.99 ± 0.01a
60	Control	0.45 ± 0.05a	8.63 ± 0.05a	0.31 ± 0.02a	0.01 ± 0.00e	25.98 ± 4.03c	24.81 ± 0.30a	14.79 ± 3.14a	2.77 ± 0.26a	0.87 ± 0.06d	0.92 ± 0.04b
	MSG‐I	0.27 ± 0.05b	8.53 ± 0.05a	0.25 ± 0.02a	0.13 ± 0.00d	27.16 ± 4.03c	23.13 ± 0.30b	15.07 ± 3.14a	2.75 ± 0.26a	1.15 ± 0.06c	0.97 ± 0.04ab
	MSG‐II	0.23 ± 0.05b	8.53 ± 0.05a	0.18 ± 0.02b	0.17 ± 0.00c	33.67 ± 4.03bc	22.90 ± 0.30b	15.50 ± 3.14a	2.84 ± 0.26a	1.38 ± 0.06b	0.99 ± 0.04ab
	MSG‐III	0.22 ± 0.05b	8.34 ± 0.05b	0.12 ± 0.02bc	0.20 ± 0.00b	42.35 ± 4.03ab	22.39 ± 0.30bc	19.07 ± 3.14a	2.91 ± 0.26a	1.51 ± 0.06ab	1.00 ± 0.04ab
	MSG‐IV	0.16 ± 0.05b	8.18 ± 0.05c	0.08 ± 0.02c	0.25 ± 0.00a	52.77 ± 4.03a	21.78 ± 0.30c	19.76 ± 3.14a	3.31 ± 0.26a	1.63 ± 0.06a	1.09 ± 0.04a

*Note*: Different lowercase letters within the same column and within the same sampling time indicate significant differences among treatment groups (*p* < 0.05) (*n* = 7*3, mean ± standard error). SOD (ng/mL), GSH‐Px (ng/mL), CAT (mU/mL), ROS (U/L), MDA (nmol/mL), NRF‐2 (ng/mL), TNF‐α (ng/L), IL‐6 (ng/L), 8‐OHdG (ng/mL) and caspase‐3 (ng/mL).

Abbreviations: IL‐6, interleukin 6; NRF‐2, nuclear factor erythroid 2‐related factor 2; ROS, reactive oxygen species; TNF‐α, tumour necrosis factor α.

## 4 DISCUSSION

In the FT‐IR spectra, the absorption bands observed in the range of 3000–3500 cm^−1^ are attributed to NH and OH groups, those in the range of 2500–3000 cm^−1^ to C–H groups, the band near 1751 cm^−1^ to ester groups, the band at 1653 cm^−1^ to amide groups, the band at 1610 cm^−1^ to lignin structures and the band at 1088 cm^−1^ to cellulose groups (Calderón et al., 2006). The presence of bands belonging to similar functional groups in all spectra indicates similar spectral properties among the samples. This suggests that the synthesized feeds are similar to those obtained from commercial companies.

In aquaculture, FCR and SGR are important factors indicating the efficiency or economy of any feeding method (Suharman et al., 2023). The present findings are consistent with those of Zhelyazkov and Stratev (2019) in the same fish species and Suharman et al. (2023) in catfish (*Hemibagrus nemurus*). It has been reported in the literature that glutamate acts as an attractant for fish, increases body weight gain and enhances feed intake by activating odour and taste stimuli in fish, and may play an important role in intestinal and systemic metabolism (Oehme et al., 2010; Yan & Qiu‐Zhou, 2006). Furthermore, glutamates have been reported to exert these beneficial effects as a precursor of nucleotide biosynthesis in rapidly dividing cells and as a source of energy for enterocytes (Yan & Qiu‐Zhou, 2006). Again, MSG‐mediated problems in leptin signalling, called leptin resistance, may have been effective in these weight increases (Chakraborty, 2019).

A significant increase in VSI and HSI values, similar to body weight, was observed in rainbow trout fed with MSG for 60 days and was found to be consistent with the literature (Kazmi et al., 2017). Studies have reported that levels of 1% or even 4% contribute to improved growth and stimulation of gastrointestinal tract development in different organisms (broilers and livestock) (Zhelyazkov & Stratev, 2019). However, in our study, when the histopathological findings were considered, although the biometric data desired for economic breeding, such as high body weight and improved feed conversion, were obtained depending on the MSG concentration, hydropic degeneration in very few cells and slight hyperaemia in the vessels in the liver tissue at the end of day 30 at high concentration of this additive (1% MSG), and degeneration in very few hepatocytes at the end of the feeding trial (day 60), were observed.

Although there may be different reasons for this damage, when we look at the MSG studies recommended at 1%–4% levels (Zhelyazkov & Stratev, 2019), we suggest that factors such as the type of organism studied, feeding time and age play important roles in the observed difference in these findings.

Although our findings were consistent with the literature in terms of growth and development, especially liver and intestinal histomap images, liver enzyme activities and haematological findings revealed that MSG‐IV feed concentration was harmful at low levels. Similar to our findings, the increased number of goblet cells in the anterior part of the fish intestinal mucosa was associated with the toxic effect of high doses of dietary MSG, and this mechanism was explained by Ladeira et al. as: ‘Goblet cells play an important role in maintaining intestinal homeostasis, and mucus secreted by GIS goblet cells acts as the front line of the host's innate defense; therefore, high numbers of these cells and high mucus secretion may be a protective response to dietary stress’. He also reported damage to the liver and gonads in mice exposed to subacute dietary MSG and adverse effects on some morphometric, physiological and inflammatory parameters in the intestines of pigs (Ladeira et al., 2021).

As is known, a decrease in RBC levels leads to a decrease in Hb concentration. These decreases may be explained by the direct toxicity of the MSG salt used in feed on RBC cells, which may have led to a reduction in the half‐life of RBC cells and/or may be attributed to its effect on RBC stem cells (Al‐Mousawi, 2017). Similarly, it suggests that MSG may cause alterations in red blood cell morphology, possibly through oxidative stress or direct toxic effects on erythropoiesis (Bukar et al., 2024). However, low Hb content may be associated with haemolysis of RBCs. Again, inhibition of enzyme activities and/or inhibition of haem synthesis through interference with iron incorporation or utilization may indicate a mechanism associated with anaemia (Osama et al., 2014). As one of the important defence mechanisms in the body, WBC can directly reflect the non‐specific immunity of the fish. WBC count is expected to decrease as a result of exposure to a toxic substance. In this study, the decrease in WBC count, especially in MSG‐IV group, reflects a reduction in non‐specific immunity in the organism, immunosuppression effect and suppression of non‐specific immune response and can be explained by the inhibition of WBC maturation (Alak et al., 2023). The higher WBC count in the MSG‐IV group compared to the MSG‐IV group with decreasing MSG concentration may be explained by increased antibody production, which helps the fish to survive, or by the removal of nutritional stress. In our findings, an increase in MCV level indicates an increase in cell size (macrocytic) and is also seen in pernicious anaemia and megaloblastic anaemia. MCH increase occurs in some types of anaemia with large cell size (macrocytic anaemia), whereas a low MCHC concentration indicates a low concentration of Hb in the blood, which is clear evidence of anaemia (Al‐Mousawi, 2017). In this study, the significant increase in MCV in the MSG groups indicates a stress response to MSG, whereas the decrease in MCHC levels is probably related to decreased Hb synthesis and swelling of erythrocytes, resulting in increased O_2_ transport capacity (Alak et al., 2023). Our findings are consistent with the literature reporting that high‐dose MSG causes macrocytosis (Bukar et al., 2024). The decrease in PLT levels during the feeding programme is consistent with the literature, suggesting that MSG‐induced oxidative stress may impair platelet production (Ajibola et al., 2012). Decrease in PLT or Hct indicates anaemia in fish (Alak et al., 2023). In our study, feeding with MSG was found to increase ESR levels, but these increases were not significant (*p* > 0.05). ESR is a common haematological test and a non‐specific method of assessing tissue damage and inflammation under stressful conditions. An increase or decrease in ESR indicates physiological dysfunctions in the fish. Stress can increase ESR due to erythrocyte fragility (Docan et al., 2018). In addition, increases in ESR may be attributed to swelling of red blood cells (Verma & Prakash, 2018). Relative changes in the indices were detected in the haematological picture mentioned above, but it has been reported in the literature that haematological disorders may be reversible under the effect of low doses of toxin (Arnaudov & Arnaudova, 2022).

Numerous adverse effects (oxidative stress, hepatotoxicity, neurotoxicity, cardiac toxicity, inflammatory response) reported in preclinical studies in the literature are controversial (Thuy et al., 2020). Discussions on this issue are probably due to the involvement of endogenous glutamate in physiological and pathological processes. Glutamate has several physiological functions: it is a precursor of important metabolites, serves as an important substrate for energy production in enterocytes, acts as a mediator in protein metabolism, functions as an oxidative stress modulator or metabolic regulator and is a central nervous system excitatory neurotransmitter (Zanfirescu et al., 2019). More recent studies have examined other metabolic and toxic effects of MSG, and a number of reports have demonstrated oxidative stress induction in different tissues of experimental animals after administration of chronic doses of MSG (Bera et al., 2017; Chakraborty, 2019).

The liver is involved in detoxification and metabolism and can be directly affected by toxic chemicals or their metabolites, such as MSG (Kazmi et al., 2017). As is well known, after oral administration, glutamate is oxidized in enterocytes of the small intestine and converted into other amino acids or used as a precursor in the synthesis of different bioactive compounds (Zanfirescu et al., 2019). This confirms that it causes oxidative stress in tissues through the formation of free radicals and alters antioxidant reactions catalysed by ROS‐scavenging enzymes (Bera et al., 2017). This image confirms the progression of oxidative damage resulting from the production of ROS (Kazmi et al., 2017). ROS can induce cellular damage by promoting cellular membrane MDA; therefore, cells require a pro‐oxidant/antioxidant balance and scavenging of ROS to maintain cell homoeostasis (García‐Medina et al., 2010).

When antioxidant function is impaired, free radicals (including free oxygen radicals, hydroxyl free radicals and nitrogen free radicals, etc.) accumulate, resulting in oxidative stress with increased ROS in the body and causing damage to cellular membranes and organelles (Jian et al., 2020). As a result of the disrupted cell structure, DNA damage occurs due to the release of toxic cations that alter cell integrity and ROS attack on the DNA structure. Loss of membrane integrity can lead to cell death (Temiz & Kargın, 2022). Our results showed that high MSG administration induced ROS production in the tissue, followed by excess ROS causing oxidative damage to DNA (increased 8‐OHdG content) and lipid peroxidation (increased MDA content). This confirms the progression of oxidative damage resulting from increased ROS production. In addition, increased levels of cytokines IL‐6 and TNF‐α were observed, along with inhibition of NRF‐2 and antioxidant enzyme activities. This indicated that MSG administration reduced the capacity to scavenge ROS in tissues and disrupted the dynamic balance between ROS production and elimination (Jian et al., 2020). Consistent with our findings, in a different study, MSG administration increased hepatic lipid peroxidation, decreased glutathione (GSH) level, inhibited CAT/SOD activity, impaired cytokine response, increased oxidative stress and MDA levels and elevated apoptotic rates (Zanfirescu et al., 2019).

The present study is the first to assess fish health and evaluate the effects of dietary MSG through a holistic approach by determining biometric changes (growth, body weight gain, feed evaluation, HSI, VSI, etc.), haematological parameters (RBC, WBC, Hg, Hct, MCV, MCH, MCHC and ESR), antioxidant/cytokine enzyme levels and histopathological monitoring; therefore, further studies are needed to elucidate the effects of MSG on aquatic organisms. In our study, the initial fish weight, the short feeding period and the effectiveness of the dosage application should not be ignored in the evaluation of our findings. However, the use of MSG is still controversial, and limited studies have been conducted in humans, particularly regarding its potential hepatotoxic, neurotoxic and genotoxic effects. Based on this phenomenon, such feeding studies in aquatic organisms are more meaningful and provide motivation for further studies using robust methodologies to assess causal links with disease.

## FUNDING INFORMATION

This study was supported with code 1919B012401247 by TÜBİTAK 2209‐A – University Student Research Projects Support Program.

## AUTHOR CONTRIBUTIONS

G. A.: Conceptualization; investigation; writing ‐ original draft; validation; methodology; visualization; writing ‐ review and editing; project administration; supervision; resources; formal analysis. F.B. and. A.A.: Conceptualization; investigation; resources; formal analysis. A.Ç.Y., S.Y., E.E.E., N.G.Y., E.M.K., M.K., T.A., E.Z.Ö., K.K., M.A.E., A.U., M.P., V.P.: Analyzed and interpreted the data, investigation; resources; formal analysis. M.A.: Writing ‐ review and editing

## Supporting information


**Table S1.** The feeding programme table.
**Table S2.** Catalogue number of oxidative stress response, proinflammatory cytokine enzyme, DNA damage and apoptosis biomarker.

## Data Availability

The data that support the findings of this study are available from the corresponding author upon reasonable request.
