# Supplementary material for: Haematological and histopathological monitoring of monosodium glutamate–supplemented feeding in rainbow trout (Oncorhynchus mykiss): A possible modelling for a single health approach
Source: J Fish Biol. 2026 Apr 28;109(2):839–48. doi: 10.1111/jfb.70460 (PMC13521728; doi:10.1111/jfb.70460)
Supplement: Supplementary file 1 [file JFB-109-839-s001.docx]

**Table S1**. **The feeding program table**

| **Fish Weight (g)** | **Water Temperature (°C)** | | | | | | | | | |
| --- | --- | --- | --- | --- | --- | --- | --- | --- | --- | --- |
|  | 8.3 | 9.4 | 10.5 | 11.7 | 12.8 | 13.9 | 15.0 | 16.1 | 17.2 | 18.3 |
| 0.53 | 3.50 | 4.29 | 5.09 | 5.88 | 7.03 | 7.87 | 8.66 | 7.87 | 7.07 | 6.68 |
| 1.3 | 2.63 | 3.22 | 3.82 | 4.41 | 5.30 | 5.90 | 6.49 | 5.90 | 5.30 | 5.01 |
| 2.6 | 2.10 | 2.58 | 3.05 | 3.53 | 4.24 | 4.72 | 5.20 | 4.72 | 4.24 | 4.01 |
| 4.6 | 2.75 | 2.15 | 2.54 | 2.94 | 3.54 | 3.93 | 4.33 | 3.93 | 3.54 | 3.34 |
| 7.5 | 1.50 | 1.84 | 2.18 | 2.52 | 3.03 | 3.37 | 3.71 | 3.37 | 3.03 | 2.86 |
| 11.4 | 1.31 | 1.61 | 1.91 | 2.21 | 2.65 | 2.95 | 3.25 | 2.95 | 2.65 | 2.50 |
| 16.4 | 1.17 | 1.43 | 1.70 | 1.96 | 2.36 | 2.62 | 2.89 | 2.62 | 2.36 | 2.23 |
| 23 | 1.05 | 1.29 | 1.53 | 1.76 | 2.00 | 2.36 | 2.60 | 2.36 | 2.12 | 2.00 |
| 31 | 0.95 | 1.17 | 1.39 | 1.60 | 1.93 | 2.15 | 2.36 | 2.15 | 1.93 | 1.82 |
| 41 | 0.88 | 1.07 | 1.27 | 1.47 | 1.67 | 1.97 | 2.16 | 1.97 | 1.77 | 1.67 |
| 52 | 0.81 | 0.99 | 1.17 | 1.36 | 1.63 | 1.82 | 2.00 | 1.82 | 1.63 | 1.54 |
| 66 | 0.75 | 0.92 | 1.09 | 1.26 | 1.52 | 1.69 | 1.86 | 1.69 | 1.52 | 1.43 |
| 81 | 0.70 | 0.86 | 1.02 | 1.18 | 1.34 | 1.57 | 1.73 | 1.57 | 1.41 | 1.34 |
| 98 | 0.66 | 0.81 | 0.95 | 1.10 | 1.33 | 1.47 | 1.62 | 1.47 | 1.33 | 1.25 |
| 120 | 0.62 | 0.76 | 090 | 1.04 | 1.25 | 1.39 | 1.53 | 1.39 | 1.25 | 1.18 |
| 142 | 0.58 | 0.72 | 0.85 | 0.98 | 1.18 | 1.31 | 1.44 | 1.31 | 1.18 | 1.11 |
| 168 | 0.55 | 0.68 | 0.80 | 0.93 | 1.12 | 1.24 | 1.37 | 1.24 | 1.12 | 1.05 |
| 197 | 0.53 | 0.64 | 0.76 | 0.88 | 1.06 | 1.18 | 1.30 | 1.18 | 1.06 | 1.00 |
| 239 | 0.50 | 0.61 | 0.73 | 0.84 | 1.01 | 1.12 | 1.24 | 1.12 | 1.01 | 0.95 |
| 267 | 0.48 | 0.59 | 0.69 | 0.80 | 0.96 | 1.07 | 1.18 | 1.07 | 0.96 | 0.91 |
| 303 | 0.46 | 0.56 | 0.66 | 0.77 | 0.92 | 1.03 | 1.13 | 1.03 | 0.92 | 0.87 |
| 350 | 0.44 | 0.54 | 0.64 | 0.74 | 0.88 | 0.98 | 1.08 | 0.98 | 0.88 | 0.83 |
| 378 | 0.42 | 0.52 | 0.61 | 0.71 | 0.85 | 0.94 | 1.04 | 0.94 | 0.85 | 0.80 |
| 413 | 0.40 | 0.50 | 0.59 | 0.68 | 0.82 | 0.91 | 1.00 | 0.91 | 0.82 | 0.77 |
| 454 | 0.39 | 0.48 | 0.57 | 0.65 | 0.79 | 0.87 | 0.96 | 0.87 | 0.79 | 0.74 |
| 504 | 0.38 | 0.46 | 0.55 | 0.63 | 0.76 | 0.84 | 0.93 | 0.84 | 0.76 | 0.72 |
| 568 | 0.36 | 0.44 | 0.53 | 0.61 | 0.73 | 0.81 | 0.90 | 0.81 | 0.73 | 0.69 |
| 650 | 0.35 | 0.43 | 0.51 | 0.59 | 0.71 | 0.79 | 0.87 | 0.79 | 0.71 | 0.67 |
| 757 | 0.34 | 0.42 | 0.49 | 0.57 | 0.68 | 0.76 | 0.84 | 0.76 | 0.68 | 0.65 |
| 908 | 0.33 | 0.40 | 0.48 | 0.55 | 0.66 | 0.74 | 0.81 | 0.74 | 0.66 | 0.63 |

**Table S2. Catalogue number of oxidative stress response, proinflammatory cytokine enzyme, DNA damage and apoptosis biomarker**

| **Oxidative stress response and proinflammatory cytokine enzyme** | | |
| --- | --- | --- |
| **Superoxide dismutase (SOD)** | ELISA kit was used to determine the level of SOD according to provider’s guide. | BT LAB, Catalogue No: EA0035FI |
| **Catalase (CAT)** | ELISA kit was used to determine the level of CAT according to provider’s guide. | BT LAB, Catalogue No: E0019Fi |
| **Glutathione peroxidase (GPx)** | ELISA kit was used to determine the level of GPx according to provider’s guide. | BT LAB, Catalogue No: E0056FI |
| **Reactive oxygen species  (ROS)** | The ROS level in fish tissues was determined by the 2′,7′-dichlorofluorescin diacetate (DCFDA) kit | ABCAM, DCFDA Cellular ROS DetectionAssay Kit, ab113851 |
| **Lipid peroxidation (MDA)** | ELISA kit was used to determine the level of MDA according to provider’s guide | BT LAB, Catalogue No: E0019FI |
| **Tumor necrosis factor alpha (TNF-α)** | For the determination of TNF-α levels (TNF-α) ELISA kit was used according to provider’s guide. | YLA0053FI, YLbiant |
| **Interleukin 6 (IL-6)** | IL-6 ELISA kit was used to determine the level of IL-6 according to provider’s guide. | YLA 0017FI, YLbiant |
| **Nuclear factor erythroid2-related factor-2 (NRF-2)** | ELISA kit was used to determine the level of NRF-2 according to provider’s guide. | BT LAB, Catalogue No: E0254FI |
| **DNA damage and apoptosis biomarker** | | |
| **8-hydroxy-2'-deoxyguanosine (8-OHdG)** | 8-OHdG levels in fish was performed according to provider’s guide | Catalogue No: 201-00-0041/SunRed |
| **Caspase-3** | Caspase-3 level (CASP3 ELISA Kit) was performed according to provider’s guide | Catalogue No: 201-00-0031) (SunRed brand) |

**Growth performance and somatic indice  formules**

*SGR = Specificgrowth rate = 100 × [ln (average fish weight at the end of the trial) − ln(average fish weight at the beginning of the trial)]/trial period,

*WG= (final weight–initial weight)×100/initial weight),

*FCR = (total dry feed intake (g) ∕ (wet weight gain(g),

*HSI = 100 × liver wet weight (g) ∕ final body weight (g)

*VSI = 100 ×viscera wet weight (g) ∕ final body weight (g)
